# Identifying Statistical Dependence in Genomic Sequences via Mutual Information Estimates

**DOI:** 10.1155/2007/14741

**Published:** 2007-12-05

**Authors:** Hasan Metin Aktulga, Ioannis Kontoyiannis, L Alex Lyznik, Lukasz Szpankowski, Ananth Y Grama, Wojciech Szpankowski

**Affiliations:** 1 Department of Computer Science, Purdue University, West Lafayette, IN 47907, USA; 2 Department of Informatics, Athens University of Economics & Business, Patission 76, Athens 10434, Greece; 3 Pioneer Hi-Breed International, Johnston, IA, USA; 4 Bioinformatics Program, University of California, San Diego, CA 92093, USA

## Abstract

Questions of understanding and quantifying the representation and amount of information in organisms have become a central part of biological research, as they potentially hold the key to fundamental advances. In this paper, we demonstrate the use of information-theoretic tools for the task of identifying segments of biomolecules (DNA or RNA) that are statistically correlated. We develop a precise and reliable methodology, based on the notion of *mutual information*, for finding and extracting statistical as well as structural dependencies. A simple threshold function is defined, and its use in quantifying the level of significance of dependencies between biological segments is explored. These tools are used in two specific applications. First, they are used for the identification of correlations between different parts of the maize zmSRp32 gene. There, we find significant dependencies between the  untranslated region in zmSRp32 and its alternatively spliced exons. This observation may indicate the presence of as-yet unknown alternative splicing mechanisms or structural scaffolds. Second, using data from the FBI's combined DNA index system (CODIS), we demonstrate that our approach is particularly well suited for the problem of discovering short tandem repeats—an application of importance in genetic profiling.

## 

[1,2,3,4,5,6,7,8,9,10,11,12,13,14,15,16,17,18,19,20,21,22]

## References

[B1] Steuer R Kurths J Daub CO Weise J Selbig J The mutual information: detecting and evaluating dependencies between variables Bioinformatics 2002 18 supplement 2 S231 S240 12386007 10.1093/bioinformatics/18.suppl_2.s231

[B2] Dawy Z Goebel B Hagenauer J Andreoli C Meitinger T Mueller JC Gene mapping and marker clustering using Shannon's mutual information IEEE/ACM Transactions on Computational Biology and Bioinformatics 2006 3 1 47 56 10.1109/TCBB.2006.9 17048392

[B3] Segal E Fondufe-Mittendorf Y Chen L A genomic code for nucleosome positioning Nature 2006 442 7104 772 778 10.1038/nature04979 16862119 PMC2623244

[B4] Osada Y Saito R Tomita M Comparative analysis of base correlations in untranslated regions of various species Gene 2006 375 1-2 80 86 16618531 10.1016/j.gene.2006.02.018

[B5] Kozak M Initiation of translation in prokaryotes and eukaryotes Gene 1999 234 2 187 208 10.1016/S0378-1119(99)00210-3 10395892

[B6] Reddy DA Mitra CK Comparative analysis of transcription start sites using mutual information Genomics, Proteomics and Bioinformatics 2006 4 3 189 195 10.1016/S1672-0229(06)60032-6 PMC5054067 17127217

[B7] Reddy DA Prasad BVLS Mitra CK Comparative analysis of core promoter region: information content from mono and dinucleotide substitution matrices Computational Biology and Chemistry 2006 30 1 58 62 10.1016/j.compbiolchem.2005.10.004 16321573

[B8] Shabalina SA Ogurtsov AY Rogozin IB Koonin EV Lipman DJ Comparative analysis of orthologous eukaryotic mRNAs: potential hidden functional signals Nucleic Acids Research 2004 32 5 1774 1782 10.1093/nar/gkh313 15031317 PMC390323

[B9] Baldi P Brunak S Frasconi P Soda G Pollastri G Exploiting the past and the future in protein secondary structure prediction Bioinformatics 1999 15 11 937 946 10.1093/bioinformatics/15.11.937 10743560

[B10] Battail G Should genetics get an information-theoretic education? Genomes as error-correcting codes IEEE Engineering in Medicine and Biology Magazine 2006 25 1 34 45 16485390 10.1109/memb.2006.1578662

[B11] Gao H Gordon-Kamm WJ Lyznik LA ASF/SF2-like maize pre-mRNA splicing factors affect splice site utilization and their transcripts are alternatively spliced Gene 2004 339 1-2 25 37 15363843 10.1016/j.gene.2004.06.047

[B12] Cover TM Thomas JA Elements of Information Theory 1991 John Wiley & Sons, New York, NY, USA

[B13] Good PI Resampling Methods 2005 Birkhäuser, Boston, Mass, USA

[B14] Manly B Randomization, Bootstrap and Monte Carlo Methods in Biology 1977 Chapman & Hall/CRC, Boca Raton, Fla, USA

[B15] Lehmann EL Romano JP Testing Statistical Hypotheses 2005 3 Springer, New York, NY, USA

[B16] Schervish MJ Theory of Statistics 1995 Springer, New York, NY, USA

[B17] Hagenauer J Dawy Z Göbel B Hanus P Mueller J Genomic analysis using methods from information theory Proceedings of IEEE Information Theory Workshop (ITW '04), San Antonio, Tex, USA, October 2004 55 59

[B18] Goebel B Dawy Z Hagenauer J Mueller JC An approximation to the distribution of finite sample size mutual information estimates Proceedings of IEEE International Conference on Communications (ICC '05), Seoul, Korea, May 2005 2 1102 1106

[B19] Hutter M Distribution of mutual information Advances in Neural Information Processing Systems 14 2002 MIT Press, Cambridge, Mass, USA 399 406

[B20] Hughes TA Regulation of gene expression by alternative untranslated regions Trends in Genetics 2006 22 3 119 122 10.1016/j.tig.2006.01.001 16430990

[B21] Åberg J Shtarkov YuM Smeets BJM Multialphabet coding with separate alphabet description Proceedings of the International Conference on Compression and Complexity of Sequences, Positano, Italy, June 1997 56 65

[B22] Orlitsky A Santhanam NP Viswanathan K Zhang J Limit results on pattern entropy IEEE Transactions on Information Theory 2006 52 7 2954 2964

